# Crystal structure of 2-[(5-amino-1-tosyl-1*H*-pyrazol-3-yl)­oxy]-1-(4-meth­oxy­phen­yl)ethan-1-one 1,4-dioxane monosolvate

**DOI:** 10.1107/S205698902301054X

**Published:** 2024-01-01

**Authors:** Nadia H. Metwally, Galal H. Elgemeie, Peter G. Jones

**Affiliations:** a Chemistry Department, Faculty of Science, Cairo University, Giza, Egypt; b Chemistry Department, Faculty of Science, Helwan University, Cairo, Egypt; c Institut für Anorganische und Analytische Chemie, Technische Universität Braunschweig, Hagenring 30, D-38106 Braunschweig, Germany; Universität Greifswald, Germany

**Keywords:** crystal structure, pyrazole, tos­yl, hydrogen bond

## Abstract

In the structure of the title compound, C_19_H_19_N_3_O_5_S·C_4_H_8_O_2_, the two independent dioxane mol­ecules each display inversion symmetry. The pyrazole ring is approximately parallel to the aromatic ring of the oxy-ethanone group and approximately perpendicular to the tolyl ring of the sulfonyl substituent. An extensive system of classical and ‘weak’ hydrogen bonds connects the residues to form a layer structure parallel to (201), within which dimeric subunits are conspicuous; neighbouring layers are connected by classical hydrogen bonds to dioxanes and by ‘weak’ hydrogen bonds from H_tol­yl_ donors.

## Abstract 2

In the mol­ecular structure of the title compound, the pyrazole and its oxy-ethanone substituent lie parallel to each other, whereas the sulfonyl ring is roughly perpendicular to the pyrazole. The residues form a layer structure by hydrogen bonding.

## 1. Chemical context

We are currently developing several synthetic strategies for the preparation of new heterocyclic compounds containing *N*-sulfonyl­amino- and *N*-sulfonyl moieties, which have recently been shown to possess significant biological activity as novel anti-covid-19, anti­microbial and anti­viral agents (Azzam *et al.*, 2019 ▸; Elgemeie *et al.*, 2019 ▸, 2022 ▸; Zhu *et al.*, 2013 ▸). Some of our recently reported *N*-aryl­sulfonyl­pyrazoles (Elgemeie *et al.*, 1998 ▸, 2002 ▸, 2013 ▸) have been used by other groups as inhibitors of NS2B-NS3 virus and cathepsin B16 (Myers *et al.*, 2007 ▸; Sidique *et al.*, 2009 ▸). In this context, we are seeking simple and innovative syntheses for other new derivatives of *N-*sulfonated pyrazoles, in the hope of finding different scaffolds for use as promising future drugs (Zhang *et al.*, 2020 ▸).

**Figure 1:**
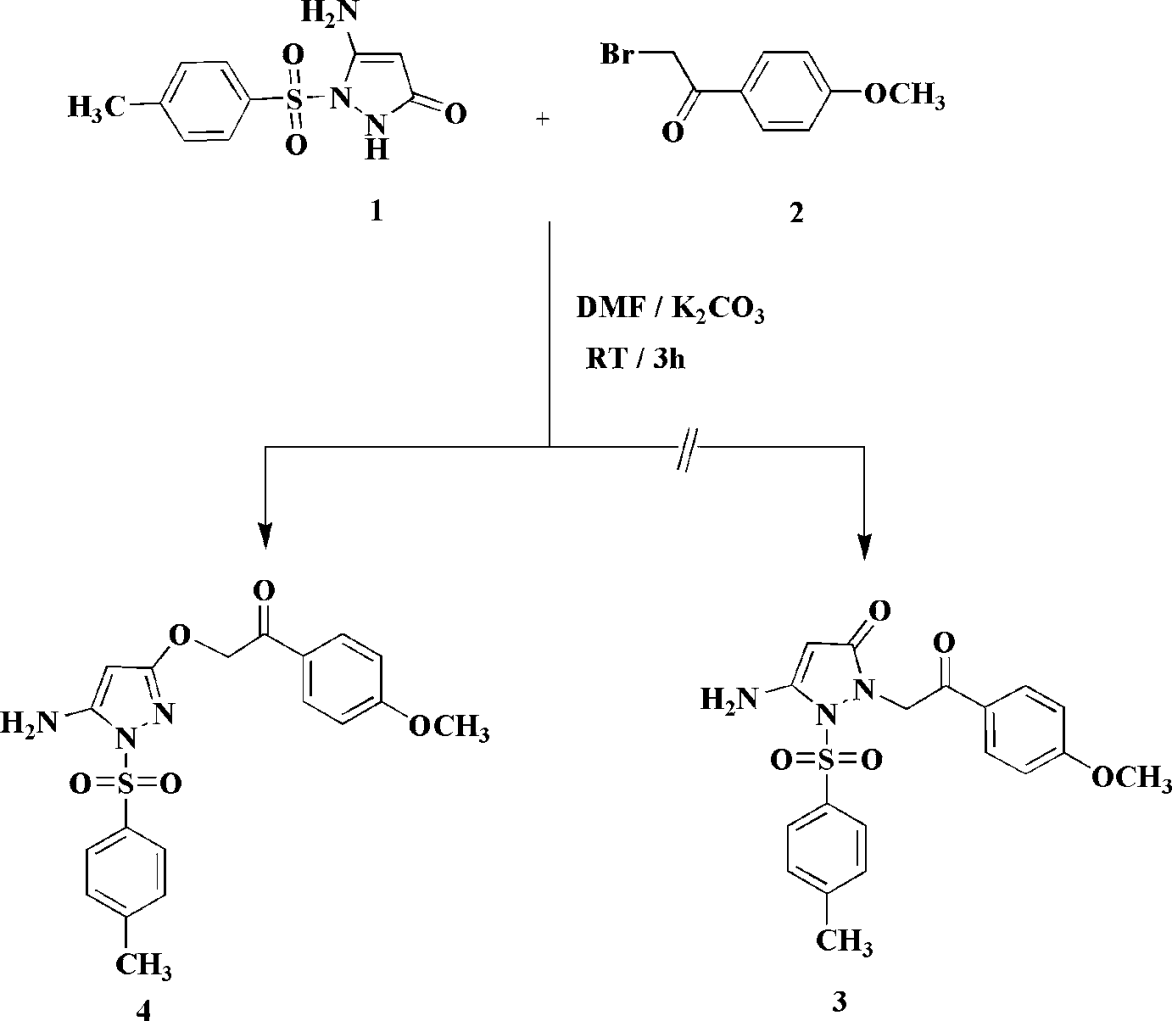
Reaction scheme for the synthesis of **4**.

We have previously prepared both *N*-alkyl­ated (Metwally *et al.*, 2021*a* ▸) and *O*-alkyl­ated (Metwally *et al.*, 2021*b* ▸) derivatives of *N*-tosyl­pyrazole **1**. In order to determine which factors lead to the formation of *N*-alkyl­ated or *O*-alkyl­ated products of *N*-tosyl­pyrazole, a reaction (Fig. 1 ▸) was conducted of *N*-tosyl­pyrazole (**1**) with 2-bromo-1-(4-meth­oxy­phen­yl)ethan-1-one (**2**) and potassium carbonate in dry *N,N*-di­methyl­formamide at room temperature. This yielded an adduct for which two isomeric structures are possible, the *O*-alkyl­ated or *N*-alkyl­ated *N*-tosyl­pyrazoles **3** or **4**. The ^1^H NMR spectrum of the product showed five singlet signals at δ = 2.37, 3.85, 4.92, 5.42 and 6.31 ppm, assigned to the CH_3_, OCH_3_, CH-pyrazole, CH_2_ and NH_2_ protons, respectively, in addition to signals from the aromatic protons. The formation of a mixture could thereby be excluded. The X-ray structure determination unambiguously confirmed the formation of the *O*-alkyl­ated *N*-sulfonyl­pyrazole **4**. The synthesis of this product rather than the isomeric *N*-tosyl­pyrazole **3** might be attributable to the possibility that **4** is the thermodynamically controlled product because of less steric hindrance. 
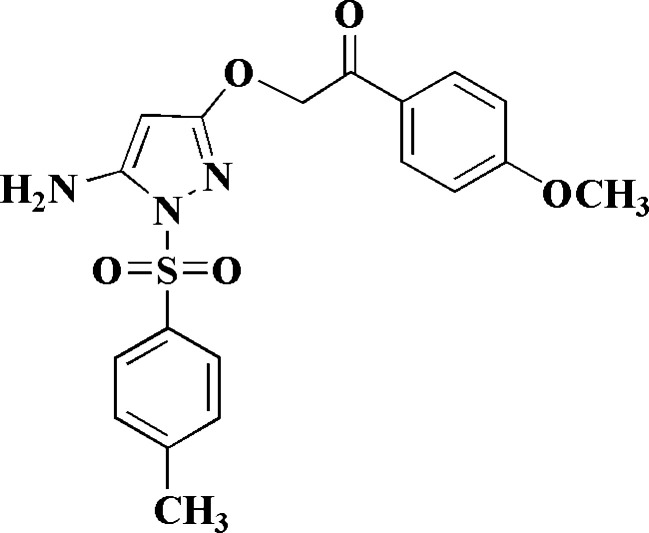


## 2. Structural commentary

**Figure 2:**
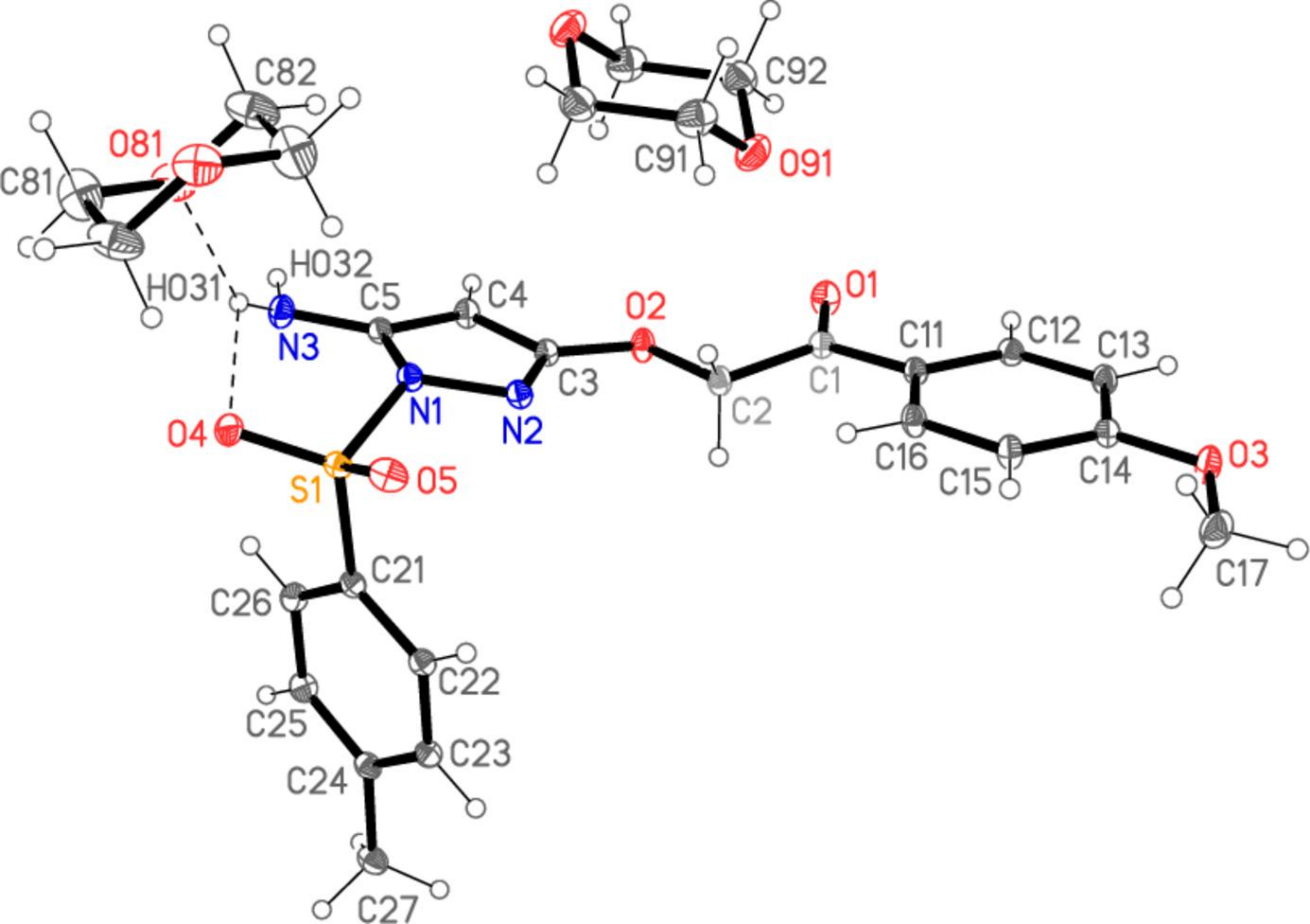
The structure of compound **4′** in the crystal. Both dioxane mol­ecules display inversion symmetry; only the asymmetric unit is numbered. Ellipsoids represent 50% probability levels. Dashed lines indicate hydrogen bonds. See also the *Refinement* section.

The structure of compound **4** (as its 1,4-dioxane solvate **4′**) is shown in Fig. 2 ▸, where the dioxane rings, which lie around inversion centres, have been completed by symmetry. The dioxanes containing O81 and O91 are henceforth referred to as dioxanes *1* and *2* respectively. A selection of mol­ecular dimensions is given in Table 1 ▸; these may be considered as normal. The atom sequence C5—C4—C3—O2—C2—C1—C11—C12 is characterized by torsion angles close to ±180°; the greatest deviation from anti­periplanar values is seen for C3—O2—C2—C1 at −166.35 (3)°. This extended anti­periplanar sequence causes the heterocycle and the ring at C11 to be approximately parallel, whereas the heterocycle and the tolyl rings are approximately perpendicular to each other [inter­planar angles of 7.58 (3) and 82.92 (1)°, respectively]. An intra­molecular hydrogen bond N3—H032⋯O4 is formed from an amino hydrogen atom to a sulfonyl oxygen atom (Table 2 ▸). The nitro­gen atom N3 of the amine group is somewhat pyramidalized; N3 lies 0.177 (5) Å outside the plane of C5, H031 and H032, and the angle sum at N3 is 350.2°.

**Figure 3:**
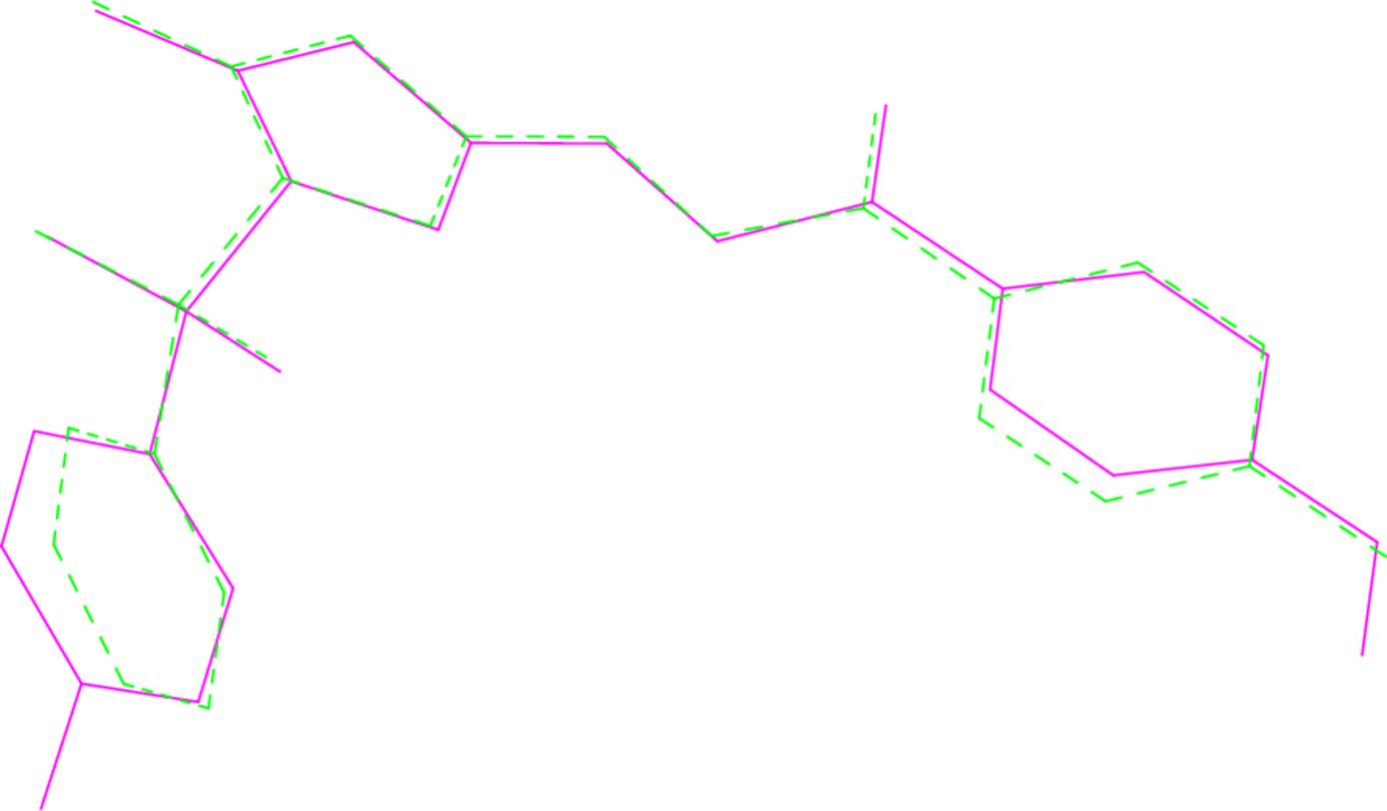
A least-squares fit of **4** and the structure of the closely related (but solvent-free) 2-[(5-amino-1-(phenyl­sulfon­yl)-1*H*-pyrazol-3-yl)­oxy]-1-(*p*-tol­yl)ethan-1-one **5** (Metwally *et al.*, 2021*b* ▸); the latter is shown with dashed bonds.

The structure of **4′** should be compared with the closely related 2-[(5-amino-1-(phenyl­sulfon­yl)-1*H*-pyrazol-3-yl)­oxy]-1-(*p*-tol­yl)ethan-1-one **5** (Metwally *et al.*, 2021*b* ▸), which has a tosyl­sulfonyl rather than a phenyl­sulfonyl group, and a 4-methyl rather than a 4-meth­oxy substituent at the other phenyl ring; this compound, however, crystallized solvent-free, so that the two structures cannot be isotypic. It forms an analogous intra­molecular hydrogen bond to that of **4′**. A least-squares fit of all non-hydrogen atoms except the differing substituents (Fig. 3 ▸), performed with *XP* (Siemens, 1994 ▸) gave an r.m.s. deviation of 0.21 Å; the ring orientation of the tosyl ring is the poorest fit [*cf.* N1—S1—C21—C22 torsion angle of 101.19 (3)° in **4′** compared to 111.54 (3)° for the corresponding angle in **5**].

## 3. Supra­molecular features

**Figure 4:**
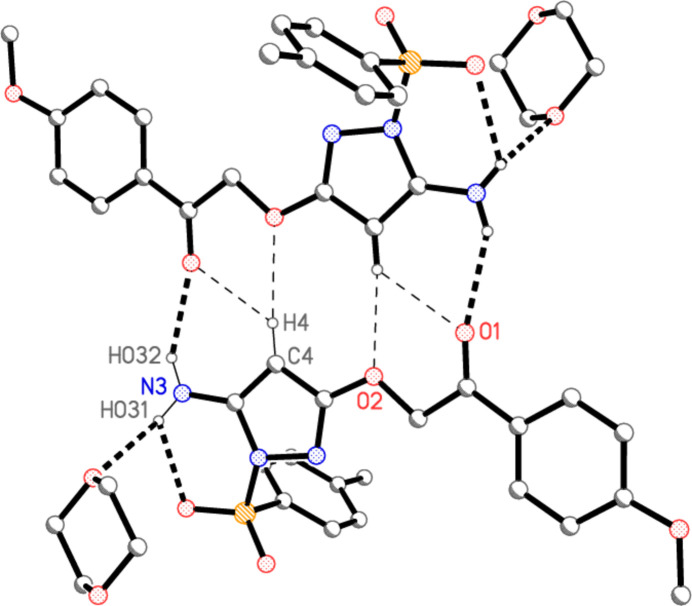
The hydrogen-bonded dimeric unit of compound **4′**. Classical and ‘weak’ hydrogen bonds are indicated by thick and thin dashed lines respectively. Hydrogen atoms not involved in these hydrogen bonds are omitted for clarity. Radii are arbitrary.

**Figure 5:**
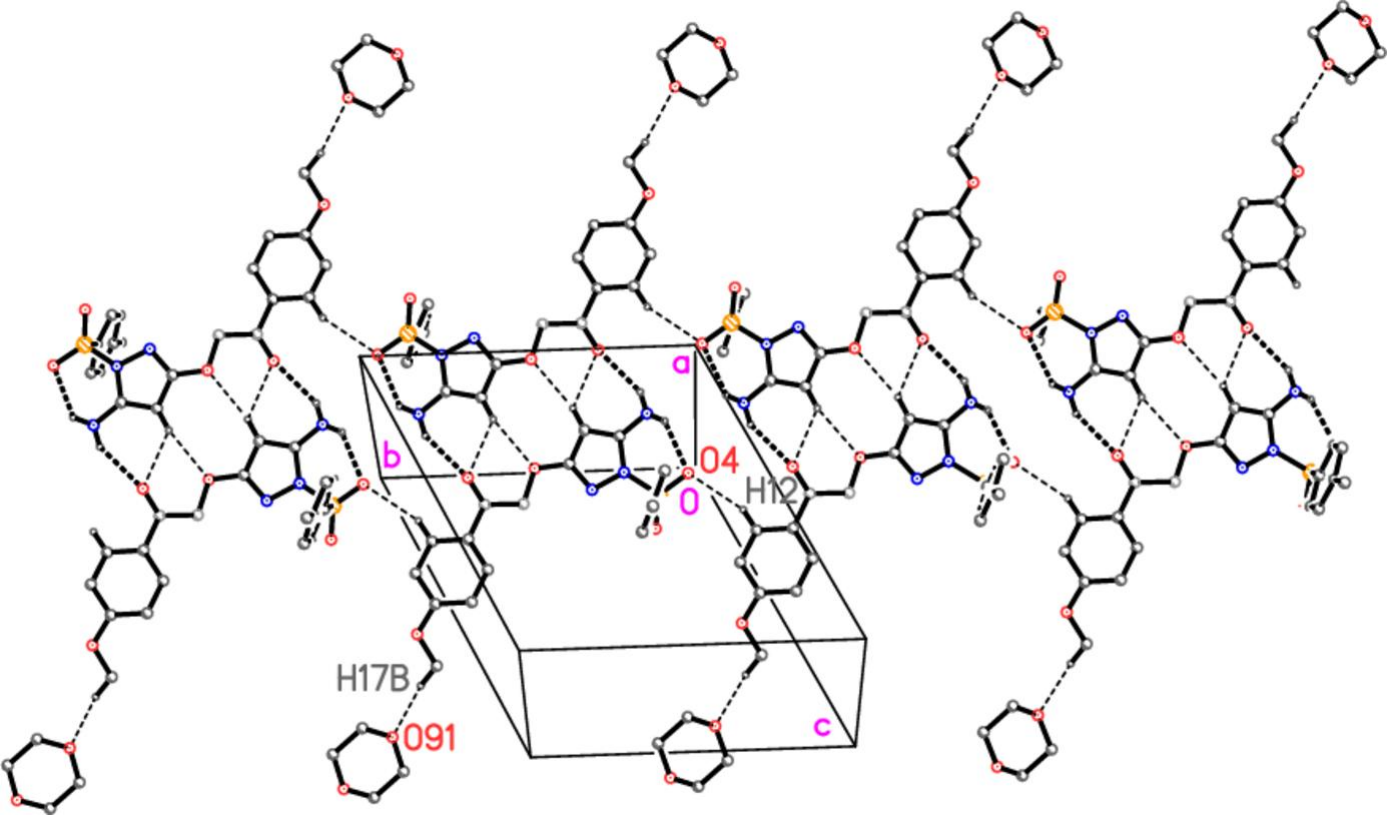
A ribbon of connected dimers of compound **4′**, viewed perpendicular to (201), with dioxanes *2*, which link to the neighbouring ribbons (not shown). Atoms that connect the dimeric units are labelled. Dioxanes *1* are omitted.

**Figure 6:**
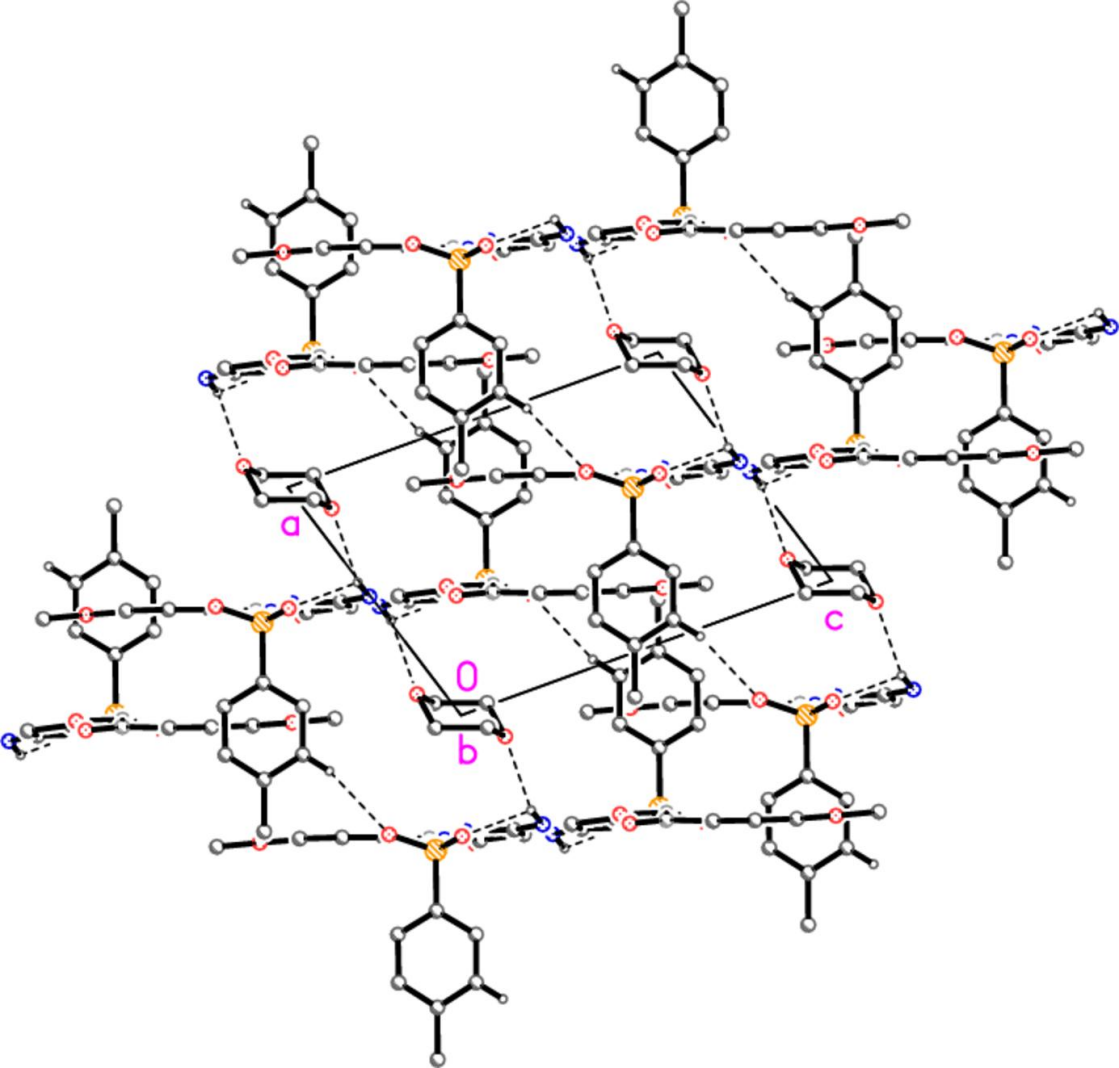
Packing of compound **4′** viewed edge-on to the layer structure (projected parallel to the *b* axis; the layers lie horizontally), thus showing the role of the dioxanes *1* and the hydrogen bond H25⋯O6 in bridging the layers. Hydrogen bonds H27*A*⋯O3, which also connect the layers, are not shown; they are formed at the points where C27 of one layer projects into the next layer and lie almost parallel to the view direction.

For details of hydrogen bonds, see Table 2 ▸. Within the asymmetric unit (Fig. 2 ▸), dioxane *1* is connected to the mol­ecule of **4** by a classical hydrogen bond N3—H031⋯O81, which is part of a three-centre system; the other branch is the intra­molecular N3—H032⋯O4. Dioxane *2* is connected by the ‘weak’ hydrogen bond C17—H17*B*⋯O91 (henceforth, we omit the description ‘weak’ for C—H⋯O inter­actions). The most striking supra­molecular feature is then the formation of inversion-symmetric dimers by the classical hydrogen bond N3—H032⋯O1 and the three-centre hydrogen bond system C4—H4⋯(O1,O2) (Fig. 4 ▸; the operator for the acceptor atoms is 1 − *x*, 1 − *y*, −*z*). The dimers are further connected to ribbons parallel to the *b* axis by the weak hydrogen bond C12—H12⋯O4 (operator −*x*, 2 − *y*, 1 − *z*), and adjacent ribbons are connected *via* dioxanes *2* by the hydrogen bond C17—H17*B*⋯O91 (operator −*x*, 2 − *y*, 1 − *z*) (Fig. 5 ▸). The translation vector between adjacent ribbons is [10 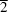 ], so that the ribbons lie in planes parallel to (201). The tolyl rings (forming the hydrogen bonds H25⋯O5 and H27*A*⋯O3) and the dioxanes *1* connect adjacent layers and are approximately perpendicular to the layers (Fig. 6 ▸).

In our previous structure (**5**; Metwally *et al.*, 2021*b* ▸), the mol­ecules also associate via hydrogen bonds N—H⋯O_carbon­yl_, to form a broad ribbon structure.

## 4. Database survey

The search employed the routine ConQuest (Bruno *et al.*, 2002 ▸), part of Version 2022.3.0 of the Cambridge Database (Groom *et al.*, 2016 ▸).

A search for pyrazole structures with the same substitution pattern as **4** (*i.e*. S at N1, O at C3, N at C5) gave only one hit (apart from **5**), namely 5-amino-1-[(4-fluoro­phen­yl)sulfon­yl]-1*H*-pyrazol-3-yl thio­phene-2-carboxyl­ate (refcode YILPUF; Myers *et al.*, 2007 ▸), in which only the O-substituent differs significantly from that of **4**. Analogously to **4**, the thio­phene ester group is approximately parallel to, and the sulfonate ring perpendicular to, the pyrazole ring. The packing of the solvent-free structure involves hydrogen bonds of the type N—H⋯O_sulfon­yl_ and N—H⋯N2_pyrazole_, which link the mol­ecules by translation to form a ribbon structure.

## 5. Synthesis and crystallization

A mixture of 5-amino-1-tosyl-1,2-di­hydro-3*H*-pyrazol-3-one **1** (0.01 mol), 2-bromo-1-(4-meth­oxy­phen­yl)ethan-1-one **2** (0.01 mol) and anhydrous potassium carbonate (0.01 mol) in *N,N*-di­methyl­formamide (5 mL) was stirred at room temperature for 3 h. The mixture was poured onto ice–water; the solid thus formed was filtered off and recrystallized from a mixture of ethanol and 1,4-dioxane to give pale brown crystals of **4′** in 75% yield, m.p. 493 K. The crystals lose 1,4-dioxane gradually on exposure to the air. IR (KBr, cm^−1^): 3468, 3366 (NH_2_), 1691 (CO); ^1^H NMR (DMSO-*d*_6_): δ = 2.37 (*s*, 3H, CH_3_), 3.85 (*s*, 3H, OCH_3_), 4.92 (*s*, 1H, CH pyrazole), 5.42 (*s*, 2H, CH_2_), 6.31 (*s*, 2H, NH_2_), 7.06 (*d*, 2H, *J* = 8.1 Hz, Ar), 7.34 (*d*, 2H, *J* = 7.8 Hz, Ar), 7.62 (*d*, 2H, *J* = 7.8 Hz, Ar), 7.92 (*d*, 2H, *J* = 8.1 Hz, Ar); ^13^C NMR (DMSO-*d*_6_): δ = 21.08, 55.55, 66.36, 69.52, 77.09, 114.05, 127.24, 129.71, 130.09, 133.19, 145.04, 159.87, 163.51, 165.78, 191.69. Analysis calculated for C_19_H_19_N_3_O_5_S (401.44); C 56.85, H 4.77, N 10.47, S 7.99. Found: C 56.6, H 4.9, N10.7, S 7.8%.

## 6. Refinement

Crystal data, data collection and structure refinement details are summarized in Table 3 ▸. Hydrogen atoms of the NH_2_ group were refined freely, but with N—H distances restrained to be approximately equal (command ‘SADI’). The methyl groups were included as idealized rigid groups allowed to rotate but not tip (command ‘AFIX 137′, with C—H = 0.98 Å, H—C—H = 109.5°; all methyl hydrogens, even those of the tosyl group, were shown clearly in the circular difference-density map). Other hydrogen atoms were included using a riding model starting from calculated positions (C—H_aromatic_ = 0.95 Å, C—H_methyl­ene_ = 0.99 Å). The *U*_iso_(H) values were fixed at 1.5 × *U*_eq_ of the parent carbon atoms for the methyl group and 1.2 × *U*_eq_ for other hydrogens. A total of six badly fitting reflections (with |error/esd| > 9.25) were removed from the refinement with ‘OMIT’ commands.

Both dioxane sites involve inversion centres. The dioxane site *2* was slightly disordered, with an occupation factor of 0.069 (2) for the minor component; in the sections above, only the major component is discussed. To improve refinement stability, appropriate restraints were employed (commands ‘SIMU’ and ‘SAME’), but the dimensions of disordered groups should always be inter­preted with caution. Furthermore, the assignment of O and C atoms to the minor site should be regarded as tentative. In Fig. 2 ▸ the dioxane *2* is centred on 0, 0.5, 0. To show its hydrogen bond H17*B*⋯O91, *2* would need to be transformed to a position centred on 0, 1.5, 0, which lies outside the unit cell.

## Supplementary Material

Crystal structure: contains datablock(s) I, global. DOI: 10.1107/S205698902301054X/yz2045sup1.cif

Structure factors: contains datablock(s) I. DOI: 10.1107/S205698902301054X/yz2045Isup2.hkl

Supporting information file. DOI: 10.1107/S205698902301054X/yz2045Isup3.cml

CCDC reference: 2313157

Additional supporting information:  crystallographic information; 3D view; checkCIF report

## Figures and Tables

**Table 1:** Selected geometric parameters (Å, °)

N1—C5	1.4044 (4)	N2—C3	1.3183 (4)
N1—N2	1.4121 (4)	C3—C4	1.4190 (5)
N1—S1	1.6737 (3)	C4—C5	1.3749 (5)
			
C5—N1—N2	111.05 (3)	N2—C3—C4	114.91 (3)
C5—N1—S1	126.29 (2)	C5—C4—C3	104.48 (3)
C3—N2—N1	102.75 (3)	C4—C5—N1	106.70 (3)
			
C11—C1—C2—O2	178.02 (3)	C2—C1—C11—C12	170.98 (3)
C1—C2—O2—C3	−166.35 (3)	C1—C11—C12—C13	−177.59 (3)
C2—O2—C3—C4	174.67 (3)	N1—S1—C21—C22	101.19 (3)
O2—C3—C4—C5	−177.41 (3)		

**Table 2:** Hydrogen-bond geometry (Å, °)

*D*—H⋯*A*	*D*—H	H⋯*A*	*D*⋯*A*	*D*—H⋯*A*
C4—H4⋯O1^i^	0.95	2.45	3.1317 (4)	129
C4—H4⋯O2^i^	0.95	2.55	3.4625 (4)	162
N3—H031⋯O4	0.87 (1)	2.28 (1)	2.8015 (5)	118 (1)
N3—H031⋯O81	0.87 (1)	2.43 (1)	3.1875 (5)	145 (1)
N3—H032⋯O1^i^	0.88 (1)	2.30 (1)	3.0867 (4)	150 (1)
C17—H17*B*⋯O91^ii^	0.98	2.49	3.4174 (6)	159
C12—H12⋯O4^iii^	0.95	2.54	3.4351 (4)	157
C25—H25⋯O5^iv^	0.95	2.59	3.4122 (4)	145
C27—H27*A*⋯O3^v^	0.98	2.46	3.3348 (5)	149

Symmetry codes: (i) 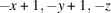 ; (ii) 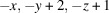 ; (iii) 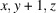 ; (iv) 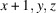 ; (v) 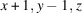 .

**Table 3:** Experimental details

Crystal data	
Chemical formula	C_19_H_19_N_3_O_5_S·C_4_H_8_O_2_
*M*_r_	489.53
Crystal system, space group	Triclinic, *P* 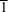
Temperature (K)	100
*a*, *b*, *c* (Å)	8.26968 (10), 12.50096 (14), 12.76743 (16)
α, β, γ (°)	116.9553 (12), 104.8418 (10), 91.0281 (10)
*V* (Å^3^)	1123.39 (3)
*Z*	2
Radiation type	Mo *K*α
μ (mm^−1^)	0.20
Crystal size (mm)	0.2 × 0.2 × 0.15

Data collection	
Diffractometer	XtaLAB Synergy
Absorption correction	Multi-scan (*CrysAlis PRO*; Rigaku OD, 2022 ▸)
*T*_min_, *T*_max_	0.914, 1.000
No. of measured, independent and observed [*I* > 2σ(*I*)] reflections	187554, 18415, 16161
*R*_int_	0.027
(sin θ/λ)_max_ (Å^−1^)	0.993

Refinement	
*R*[*F* ^2^ > 2σ(*F* ^2^)], *wR*(*F* ^2^), *S*	0.027, 0.087, 1.04
No. of reflections	18415
No. of parameters	330
No. of restraints	22
H-atom treatment	H atoms treated by a mixture of independent and constrained refinement
Δρ_max_, Δρ_min_ (e Å^−3^)	0.67, −0.38

Computer programs: *CrysAlis PRO* (Rigaku OD, 2022 ▸), *SHELXT* (Sheldrick, 2015*b* ▸), *SHELXL2019/3* (Sheldrick, 2015*a* ▸) and *XP* (Siemens, 1994 ▸).

## supplementary crystallographic information

### Crystal data

**Table:**

C_19_H_19_N_3_O_5_S·C_4_H_8_O_2_	*Z* = 2
*M**_r_* = 489.53	*F*(000) = 516
Triclinic, *P*1	*D*_x_ = 1.447 Mg m^−^^3^
*a* = 8.26968 (10) Å	Mo *K*α radiation, λ = 0.71073 Å
*b* = 12.50096 (14) Å	Cell parameters from 105453 reflections
*c* = 12.76743 (16) Å	θ = 2.6–45.0°
α = 116.9553 (12)°	µ = 0.20 mm^−^^1^
β = 104.8418 (10)°	*T* = 100 K
γ = 91.0281 (10)°	Block, colourless
*V* = 1123.39 (3) Å^3^	0.2 × 0.2 × 0.15 mm

### Data collection

**Table:**

XtaLAB Synergy diffractometer	18415 independent reflections
Radiation source: micro-focus sealed X-ray tube	16161 reflections with *I* > 2σ(*I*)
Detector resolution: 10.0000 pixels mm^-1^	*R*_int_ = 0.027
ω scans	θ_max_ = 44.9°, θ_min_ = 2.6°
Absorption correction: multi-scan (CrysAlisPro; Rigaku OD, 2022)	*h* = −16→16
*T*_min_ = 0.914, *T*_max_ = 1.000	*k* = −24→24
187554 measured reflections	*l* = −25→25

### Refinement

**Table:**

Refinement on *F*^2^	Primary atom site location: dual
Least-squares matrix: full	Hydrogen site location: mixed
*R*[*F*^2^ > 2σ(*F*^2^)] = 0.027	H atoms treated by a mixture of independent and constrained refinement
*wR*(*F*^2^) = 0.087	*w* = 1/[σ^2^(*F*_o_^2^) + (0.0517*P*)^2^ + 0.0976*P*] where *P* = (*F*_o_^2^ + 2*F*_c_^2^)/3
*S* = 1.04	(Δ/σ)_max_ = 0.002
18415 reflections	Δρ_max_ = 0.67 e Å^−^^3^
330 parameters	Δρ_min_ = −0.38 e Å^−^^3^
22 restraints	

### Special details

**Table:**

Geometry. All esds (except the esd in the dihedral angle between two l.s. planes) are estimated using the full covariance matrix. The cell esds are taken into account individually in the estimation of esds in distances, angles and torsion angles; correlations between esds in cell parameters are only used when they are defined by crystal symmetry. An approximate (isotropic) treatment of cell esds is used for estimating esds involving l.s. planes.

### Fractional atomic coordinates and isotropic or equivalent isotropic displacement parameters (Å^2^)

**Table:**

	*x*	*y*	*z*	*U*_iso_*/*U*_eq_	Occ. (<1)
C1	0.38399 (4)	0.77886 (3)	0.26518 (3)	0.01184 (4)	
C2	0.40834 (5)	0.66456 (3)	0.27750 (3)	0.01258 (5)	
H2A	0.494861	0.684712	0.356158	0.015*	
H2B	0.300694	0.628434	0.277334	0.015*	
O1	0.41714 (4)	0.79016 (3)	0.18236 (3)	0.01691 (5)	
O2	0.46134 (4)	0.58006 (3)	0.17697 (3)	0.01403 (4)	
N1	0.40343 (4)	0.30638 (3)	0.17661 (3)	0.01166 (4)	
N2	0.40577 (4)	0.43334 (3)	0.23487 (3)	0.01164 (4)	
C3	0.45301 (4)	0.46582 (3)	0.16098 (3)	0.01109 (4)	
C4	0.48807 (5)	0.36967 (3)	0.05888 (3)	0.01274 (5)	
H4	0.523520	0.374279	−0.004293	0.015*	
C5	0.45919 (4)	0.26833 (3)	0.07193 (3)	0.01129 (4)	
N3	0.47670 (5)	0.15066 (3)	0.00274 (3)	0.01555 (5)	
H031	0.4134 (12)	0.0953 (9)	0.0048 (9)	0.029 (2)*	
H032	0.4995 (12)	0.1375 (9)	−0.0649 (8)	0.024 (2)*	
C11	0.32316 (4)	0.87608 (3)	0.35963 (3)	0.01120 (4)	
C12	0.32181 (4)	0.98963 (3)	0.36261 (3)	0.01213 (5)	
H12	0.355545	1.001617	0.302459	0.015*	
C13	0.27183 (5)	1.08435 (3)	0.45230 (3)	0.01318 (5)	
H13	0.269231	1.160437	0.452620	0.016*	
C14	0.22495 (4)	1.06787 (3)	0.54284 (3)	0.01214 (5)	
C15	0.22215 (5)	0.95479 (3)	0.53954 (3)	0.01378 (5)	
H15	0.187272	0.942595	0.599186	0.017*	
C16	0.27095 (5)	0.86004 (3)	0.44803 (3)	0.01366 (5)	
H16	0.268743	0.782921	0.445597	0.016*	
O3	0.18634 (4)	1.16720 (3)	0.63073 (3)	0.01711 (5)	
C17	0.14669 (6)	1.15468 (4)	0.72774 (4)	0.01942 (6)	
H17A	0.047427	1.091958	0.692996	0.029*	
H17B	0.122138	1.232228	0.785483	0.029*	
H17C	0.243352	1.131273	0.771163	0.029*	
S1	0.43246 (2)	0.24477 (2)	0.27095 (2)	0.01097 (2)	
O4	0.41689 (4)	0.11676 (3)	0.19275 (3)	0.01594 (5)	
O5	0.31959 (4)	0.29146 (3)	0.34374 (3)	0.01605 (5)	
C21	0.64132 (4)	0.29968 (3)	0.36666 (3)	0.01082 (4)	
C22	0.67431 (4)	0.39426 (3)	0.48634 (3)	0.01175 (4)	
H22	0.584626	0.431238	0.515766	0.014*	
C23	0.84106 (4)	0.43351 (3)	0.56194 (3)	0.01265 (5)	
H23	0.864873	0.498109	0.643528	0.015*	
C24	0.97428 (4)	0.37942 (3)	0.51979 (3)	0.01249 (5)	
C25	0.93733 (5)	0.28592 (3)	0.39821 (4)	0.01400 (5)	
H25	1.027019	0.249828	0.368074	0.017*	
C26	0.77178 (4)	0.24513 (3)	0.32102 (3)	0.01313 (5)	
H26	0.747761	0.181499	0.238932	0.016*	
C27	1.15349 (5)	0.41935 (4)	0.60318 (4)	0.01590 (5)	
H27A	1.177536	0.369938	0.645265	0.024*	
H27B	1.168027	0.505056	0.664270	0.024*	
H27C	1.231683	0.409081	0.554338	0.024*	
O81	0.12771 (5)	−0.00966 (4)	−0.05908 (4)	0.02415 (7)	
C81	0.07517 (8)	−0.10432 (6)	−0.03646 (7)	0.03059 (11)	
H81A	0.171885	−0.146721	−0.022534	0.037*	
H81B	−0.015447	−0.164162	−0.109485	0.037*	
C82	−0.01034 (7)	0.05411 (7)	−0.07474 (6)	0.02876 (10)	
H82A	−0.103225	−0.001640	−0.148717	0.035*	
H82B	0.027099	0.121680	−0.087683	0.035*	
O91	0.04584 (6)	0.61849 (4)	0.10107 (4)	0.02249 (9)	0.931 (2)
C91	−0.07460 (7)	0.52971 (6)	0.09340 (5)	0.02309 (11)	0.931 (2)
H91A	−0.190244	0.538072	0.054417	0.028*	0.931 (2)
H91B	−0.068248	0.543917	0.177303	0.028*	0.931 (2)
C92	0.04101 (7)	0.59715 (5)	−0.01928 (6)	0.02259 (10)	0.931 (2)
H92A	0.127446	0.657086	−0.013383	0.027*	0.931 (2)
H92B	−0.071504	0.607473	−0.061132	0.027*	0.931 (2)
O91'	0.0276 (9)	0.5939 (7)	0.1194 (7)	0.0265 (13)*	0.069 (2)
C91'	−0.0678 (12)	0.4761 (10)	0.0740 (9)	0.0297 (17)*	0.069 (2)
H91C	−0.050956	0.455740	0.142058	0.036*	0.069 (2)
H91D	−0.189840	0.478789	0.044301	0.036*	0.069 (2)
C92'	0.0187 (13)	0.6148 (10)	0.0222 (10)	0.0312 (18)*	0.069 (2)
H92C	0.091514	0.692745	0.052555	0.037*	0.069 (2)
H92D	−0.099241	0.623833	−0.011040	0.037*	0.069 (2)

### Atomic displacement parameters (Å^2^)

**Table:**

	*U*^11^	*U*^22^	*U*^33^	*U*^12^	*U*^13^	*U*^23^
C1	0.01442 (11)	0.01135 (10)	0.01179 (10)	0.00285 (9)	0.00588 (9)	0.00610 (9)
C2	0.01679 (12)	0.01137 (11)	0.01192 (10)	0.00442 (9)	0.00707 (9)	0.00592 (9)
O1	0.02551 (13)	0.01563 (11)	0.01559 (10)	0.00521 (9)	0.01201 (10)	0.00921 (9)
O2	0.02105 (11)	0.01078 (9)	0.01466 (9)	0.00525 (8)	0.01076 (9)	0.00670 (8)
N1	0.01493 (10)	0.01194 (10)	0.01105 (9)	0.00369 (8)	0.00563 (8)	0.00690 (8)
N2	0.01435 (10)	0.01179 (10)	0.01129 (9)	0.00381 (8)	0.00585 (8)	0.00643 (8)
C3	0.01304 (10)	0.01109 (10)	0.01116 (10)	0.00323 (8)	0.00541 (8)	0.00594 (8)
C4	0.01717 (12)	0.01201 (11)	0.01200 (11)	0.00383 (9)	0.00776 (9)	0.00628 (9)
C5	0.01295 (11)	0.01167 (10)	0.01045 (10)	0.00282 (8)	0.00444 (8)	0.00569 (8)
N3	0.02191 (13)	0.01141 (10)	0.01416 (11)	0.00400 (9)	0.00808 (10)	0.00526 (9)
C11	0.01324 (11)	0.01081 (10)	0.01189 (10)	0.00300 (8)	0.00540 (9)	0.00641 (9)
C12	0.01438 (11)	0.01145 (11)	0.01278 (11)	0.00217 (9)	0.00468 (9)	0.00725 (9)
C13	0.01621 (12)	0.01050 (10)	0.01455 (11)	0.00268 (9)	0.00521 (10)	0.00700 (9)
C14	0.01351 (11)	0.01032 (10)	0.01294 (11)	0.00290 (8)	0.00475 (9)	0.00532 (9)
C15	0.01852 (13)	0.01216 (11)	0.01507 (12)	0.00501 (10)	0.00930 (10)	0.00776 (10)
C16	0.01935 (13)	0.01153 (11)	0.01547 (12)	0.00551 (10)	0.00982 (10)	0.00828 (10)
O3	0.02369 (13)	0.01157 (9)	0.01659 (11)	0.00557 (9)	0.00988 (10)	0.00497 (8)
C17	0.02350 (16)	0.01854 (15)	0.01523 (13)	0.00552 (13)	0.00945 (12)	0.00517 (12)
S1	0.01077 (3)	0.01276 (3)	0.01195 (3)	0.00076 (2)	0.00380 (2)	0.00779 (3)
O4	0.01896 (11)	0.01203 (9)	0.01620 (10)	−0.00143 (8)	0.00272 (9)	0.00760 (8)
O5	0.01293 (9)	0.02379 (13)	0.01726 (11)	0.00318 (9)	0.00781 (8)	0.01279 (10)
C21	0.01101 (10)	0.01183 (10)	0.01159 (10)	0.00221 (8)	0.00432 (8)	0.00666 (9)
C22	0.01253 (10)	0.01267 (11)	0.01202 (10)	0.00323 (8)	0.00491 (8)	0.00677 (9)
C23	0.01372 (11)	0.01260 (11)	0.01227 (11)	0.00202 (9)	0.00366 (9)	0.00652 (9)
C24	0.01179 (10)	0.01305 (11)	0.01505 (11)	0.00145 (9)	0.00357 (9)	0.00888 (10)
C25	0.01213 (11)	0.01514 (12)	0.01644 (12)	0.00372 (9)	0.00603 (9)	0.00792 (10)
C26	0.01300 (11)	0.01351 (11)	0.01350 (11)	0.00308 (9)	0.00568 (9)	0.00597 (9)
C27	0.01265 (11)	0.01752 (13)	0.01942 (14)	0.00062 (10)	0.00188 (10)	0.01186 (12)
O81	0.01741 (12)	0.03412 (18)	0.02501 (15)	−0.00010 (12)	0.00789 (11)	0.01670 (14)
C81	0.0258 (2)	0.0336 (3)	0.0431 (3)	0.00625 (19)	0.0175 (2)	0.0232 (2)
C82	0.02010 (17)	0.0471 (3)	0.0310 (2)	0.00227 (18)	0.00602 (16)	0.0293 (2)
O91	0.02279 (16)	0.01833 (15)	0.01779 (15)	0.00159 (12)	0.00519 (12)	0.00195 (12)
C91	0.02285 (19)	0.0278 (3)	0.01701 (17)	0.00463 (16)	0.00868 (14)	0.00778 (16)
C92	0.02260 (19)	0.0223 (2)	0.0241 (2)	0.00333 (15)	0.00477 (16)	0.01313 (17)

### Geometric parameters (Å, º)

**Table:**

C1—O1	1.2229 (4)	C21—C22	1.3944 (5)
C1—C11	1.4793 (5)	C21—C26	1.3988 (5)
C1—C2	1.5182 (5)	C22—C23	1.3923 (5)
C2—O2	1.4251 (4)	C22—H22	0.9500
C2—H2A	0.9900	C23—C24	1.4004 (5)
C2—H2B	0.9900	C23—H23	0.9500
O2—C3	1.3457 (4)	C24—C25	1.4030 (5)
N1—C5	1.4044 (4)	C24—C27	1.5042 (5)
N1—N2	1.4121 (4)	C25—C26	1.3906 (5)
N1—S1	1.6737 (3)	C25—H25	0.9500
N2—C3	1.3183 (4)	C26—H26	0.9500
C3—C4	1.4190 (5)	C27—H27A	0.9800
C4—C5	1.3749 (5)	C27—H27B	0.9800
C4—H4	0.9500	C27—H27C	0.9800
C5—N3	1.3639 (5)	O81—C81	1.4241 (7)
N3—H031	0.874 (9)	O81—C82	1.4279 (7)
N3—H032	0.875 (9)	C81—C82^i^	1.5137 (9)
C11—C16	1.3973 (5)	C81—H81A	0.9900
C11—C12	1.4030 (5)	C81—H81B	0.9900
C12—C13	1.3839 (5)	C82—H82A	0.9900
C12—H12	0.9500	C82—H82B	0.9900
C13—C14	1.4040 (5)	O91—C92	1.4249 (8)
C13—H13	0.9500	O91—C91	1.4301 (8)
C14—O3	1.3555 (4)	C91—C92^ii^	1.5091 (8)
C14—C15	1.3947 (5)	C91—H91A	0.9900
C15—C16	1.3911 (5)	C91—H91B	0.9900
C15—H15	0.9500	C92—H92A	0.9900
C16—H16	0.9500	C92—H92B	0.9900
O3—C17	1.4310 (5)	O91'—C92'	1.364 (11)
C17—H17A	0.9800	O91'—C91'	1.441 (11)
C17—H17B	0.9800	C91'—C92'^ii^	1.404 (15)
C17—H17C	0.9800	C91'—H91C	0.9900
S1—O5	1.4310 (3)	C91'—H91D	0.9900
S1—O4	1.4326 (3)	C92'—H92C	0.9900
S1—C21	1.7502 (3)	C92'—H92D	0.9900
			
O1—C1—C11	122.11 (3)	C23—C22—C21	118.80 (3)
O1—C1—C2	121.06 (3)	C23—C22—H22	120.6
C11—C1—C2	116.81 (3)	C21—C22—H22	120.6
O2—C2—C1	108.97 (3)	C22—C23—C24	121.14 (3)
O2—C2—H2A	109.9	C22—C23—H23	119.4
C1—C2—H2A	109.9	C24—C23—H23	119.4
O2—C2—H2B	109.9	C23—C24—C25	118.70 (3)
C1—C2—H2B	109.9	C23—C24—C27	120.92 (3)
H2A—C2—H2B	108.3	C25—C24—C27	120.38 (3)
C3—O2—C2	115.38 (3)	C26—C25—C24	121.18 (3)
C5—N1—N2	111.05 (3)	C26—C25—H25	119.4
C5—N1—S1	126.29 (2)	C24—C25—H25	119.4
N2—N1—S1	114.68 (2)	C25—C26—C21	118.67 (3)
C3—N2—N1	102.75 (3)	C25—C26—H26	120.7
N2—C3—O2	123.26 (3)	C21—C26—H26	120.7
N2—C3—C4	114.91 (3)	C24—C27—H27A	109.5
O2—C3—C4	121.78 (3)	C24—C27—H27B	109.5
C5—C4—C3	104.48 (3)	H27A—C27—H27B	109.5
C5—C4—H4	127.8	C24—C27—H27C	109.5
C3—C4—H4	127.8	H27A—C27—H27C	109.5
N3—C5—C4	130.68 (3)	H27B—C27—H27C	109.5
N3—C5—N1	122.63 (3)	C81—O81—C82	109.31 (4)
C4—C5—N1	106.70 (3)	O81—C81—C82^i^	110.95 (5)
C5—N3—H031	116.3 (6)	O81—C81—H81A	109.4
C5—N3—H032	113.2 (6)	C82^i^—C81—H81A	109.4
H031—N3—H032	121.0 (9)	O81—C81—H81B	109.4
C16—C11—C12	118.70 (3)	C82^i^—C81—H81B	109.4
C16—C11—C1	122.44 (3)	H81A—C81—H81B	108.0
C12—C11—C1	118.84 (3)	O81—C82—C81^i^	111.17 (4)
C13—C12—C11	120.65 (3)	O81—C82—H82A	109.4
C13—C12—H12	119.7	C81^i^—C82—H82A	109.4
C11—C12—H12	119.7	O81—C82—H82B	109.4
C12—C13—C14	119.93 (3)	C81^i^—C82—H82B	109.4
C12—C13—H13	120.0	H82A—C82—H82B	108.0
C14—C13—H13	120.0	C92—O91—C91	110.01 (4)
O3—C14—C15	123.98 (3)	O91—C91—C92^ii^	110.96 (4)
O3—C14—C13	115.95 (3)	O91—C91—H91A	109.4
C15—C14—C13	120.06 (3)	C92^ii^—C91—H91A	109.4
C16—C15—C14	119.33 (3)	O91—C91—H91B	109.4
C16—C15—H15	120.3	C92^ii^—C91—H91B	109.4
C14—C15—H15	120.3	H91A—C91—H91B	108.0
C15—C16—C11	121.28 (3)	O91—C92—C91^ii^	110.33 (4)
C15—C16—H16	119.4	O91—C92—H92A	109.6
C11—C16—H16	119.4	C91^ii^—C92—H92A	109.6
C14—O3—C17	117.13 (3)	O91—C92—H92B	109.6
O3—C17—H17A	109.5	C91^ii^—C92—H92B	109.6
O3—C17—H17B	109.5	H92A—C92—H92B	108.1
H17A—C17—H17B	109.5	C92'—O91'—C91'	108.6 (7)
O3—C17—H17C	109.5	C92'^ii^—C91'—O91'	111.9 (8)
H17A—C17—H17C	109.5	C92'^ii^—C91'—H91C	109.2
H17B—C17—H17C	109.5	O91'—C91'—H91C	109.2
O5—S1—O4	120.39 (2)	C92'^ii^—C91'—H91D	109.2
O5—S1—N1	106.165 (17)	O91'—C91'—H91D	109.2
O4—S1—N1	105.240 (17)	H91C—C91'—H91D	107.9
O5—S1—C21	108.966 (18)	O91'—C92'—H92C	108.5
O4—S1—C21	108.862 (18)	C91'^ii^—C92'—H92C	108.5
N1—S1—C21	106.306 (16)	O91'—C92'—H92D	108.5
C22—C21—C26	121.49 (3)	C91'^ii^—C92'—H92D	108.5
C22—C21—S1	119.94 (3)	H92C—C92'—H92D	107.5
C26—C21—S1	118.56 (3)		
			
O1—C1—C2—O2	−3.58 (5)	C15—C14—O3—C17	2.39 (6)
C11—C1—C2—O2	178.02 (3)	C13—C14—O3—C17	−176.83 (4)
C1—C2—O2—C3	−166.35 (3)	C5—N1—S1—O5	−165.61 (3)
C5—N1—N2—C3	3.25 (4)	N2—N1—S1—O5	48.49 (3)
S1—N1—N2—C3	154.29 (2)	C5—N1—S1—O4	−36.94 (4)
N1—N2—C3—O2	175.41 (3)	N2—N1—S1—O4	177.16 (3)
N1—N2—C3—C4	−1.98 (4)	C5—N1—S1—C21	78.45 (3)
C2—O2—C3—N2	−2.54 (5)	N2—N1—S1—C21	−67.45 (3)
C2—O2—C3—C4	174.67 (3)	O5—S1—C21—C22	−12.84 (3)
N2—C3—C4—C5	0.01 (4)	O4—S1—C21—C22	−145.89 (3)
O2—C3—C4—C5	−177.41 (3)	N1—S1—C21—C22	101.19 (3)
C3—C4—C5—N3	−178.08 (4)	O5—S1—C21—C26	165.95 (3)
C3—C4—C5—N1	2.01 (4)	O4—S1—C21—C26	32.90 (3)
N2—N1—C5—N3	176.69 (3)	N1—S1—C21—C26	−80.02 (3)
S1—N1—C5—N3	29.78 (5)	C26—C21—C22—C23	−0.89 (5)
N2—N1—C5—C4	−3.38 (4)	S1—C21—C22—C23	177.87 (3)
S1—N1—C5—C4	−150.30 (3)	C21—C22—C23—C24	−0.25 (5)
O1—C1—C11—C16	174.25 (4)	C22—C23—C24—C25	1.37 (5)
C2—C1—C11—C16	−7.36 (5)	C22—C23—C24—C27	−177.93 (3)
O1—C1—C11—C12	−7.40 (6)	C23—C24—C25—C26	−1.40 (5)
C2—C1—C11—C12	170.98 (3)	C27—C24—C25—C26	177.90 (3)
C16—C11—C12—C13	0.82 (5)	C24—C25—C26—C21	0.32 (5)
C1—C11—C12—C13	−177.59 (3)	C22—C21—C26—C25	0.85 (5)
C11—C12—C13—C14	1.22 (5)	S1—C21—C26—C25	−177.92 (3)
C12—C13—C14—O3	176.66 (3)	C82—O81—C81—C82^i^	−57.05 (7)
C12—C13—C14—C15	−2.59 (6)	C81—O81—C82—C81^i^	57.18 (7)
O3—C14—C15—C16	−177.31 (4)	C92—O91—C91—C92^ii^	57.63 (6)
C13—C14—C15—C16	1.88 (6)	C91—O91—C92—C91^ii^	−57.25 (6)
C14—C15—C16—C11	0.19 (6)	C92'—O91'—C91'—C92'^ii^	52.2 (12)
C12—C11—C16—C15	−1.54 (6)	C91'—O91'—C92'—C91'^ii^	−53.9 (12)
C1—C11—C16—C15	176.81 (4)		

Symmetry codes: (i) −*x*, −*y*, −*z*; (ii) −*x*, −*y*+1, −*z*.

### Hydrogen-bond geometry (Å, º)

**Table:**

*D*—H···*A*	*D*—H	H···*A*	*D*···*A*	*D*—H···*A*
C4—H4···O1^iii^	0.95	2.45	3.1317 (4)	129
C4—H4···O2^iii^	0.95	2.55	3.4625 (4)	162
N3—H031···O4	0.87 (1)	2.28 (1)	2.8015 (5)	118 (1)
N3—H031···O81	0.87 (1)	2.43 (1)	3.1875 (5)	145 (1)
N3—H032···O1^iii^	0.88 (1)	2.30 (1)	3.0867 (4)	150 (1)
C17—H17*B*···O91^iv^	0.98	2.49	3.4174 (6)	159
C12—H12···O4^v^	0.95	2.54	3.4351 (4)	157
C25—H25···O5^vi^	0.95	2.59	3.4122 (4)	145
C27—H27*A*···O3^vii^	0.98	2.46	3.3348 (5)	149

Symmetry codes: (iii) −*x*+1, −*y*+1, −*z*; (iv) −*x*, −*y*+2, −*z*+1; (v) *x*, *y*+1, *z*; (vi) *x*+1, *y*, *z*; (vii) *x*+1, *y*−1, *z*.
